# Enterococcus faecalis requires unsaturated fatty acids to overcome toxicity of environmental saturated fatty acids

**DOI:** 10.1099/mic.0.001602

**Published:** 2025-09-03

**Authors:** Qi Zou, Huijuan Dong, John E. Cronan

**Affiliations:** 1 Department of Microbiology, University of Illinois at Urbana-Champaign, Urbana, Illinois, USA; 2 Department of Biochemistry, University of Illinois at Urbana-Champaign, Urbana, Illinois, USA

**Keywords:** acyl carrier proteins, acyl phosphates, *Enterococcus faecalis*, fatty acid synthesis, phospholipid synthesis, streptococci

## Abstract

*Enterococcus faecalis* synthesizes phospholipids from either *de novo* synthesized or exogenous fatty acids. However, environmental saturated fatty acids are toxic to *E. faecalis*. The mechanism of toxicity is unknown. We report that saturated acids block growth by efficiently repressing transcription of the fatty acid biosynthesis (*fab*) genes, resulting in blockage of the synthesis of unsaturated fatty acyl chains. Saturated fatty acid toxicity depends on the chain length of the acyl chains. Growth was restored in the presence of toxic saturated fatty acids by the increased *de novo* unsaturated fatty acid synthesis, resulting from the deletion of the *fabT* gene, the repressor that regulates (*fab*) gene transcription. The addition of unsaturated fatty acids to the medium also restored growth in the presence of toxic saturated fatty acids. Overexpression of AcpA, the fatty acid synthesis acyl carrier protein, also gave increased *de novo* synthesis of unsaturated fatty acids and restored growth.

## Data Availability

Growth of the opportunistic pathogen *Enterococcus faecalis* is inhibited by exogenous saturated fatty acids. This report demonstrates that growth inhibition is due to blocking the synthesis of unsaturated fatty acids essential for functional membrane phospholipids by acyl-acyl carrier proteins (ACPs) with saturated acyl chains. These saturated acyl-ACPs bind the FabT transcriptional repressor protein and increase the affinity of FabT for the promoters of the fatty acid synthesis genes, thereby blocking the synthesis of unsaturated fatty acyl chains. Increasing fatty acid synthesis by inactivation of FabT or by blocking conversion of exogenous saturated fatty acids to acyl-ACPs reverses growth inhibition by exogenous saturated fatty acids. Addition of unsaturated fatty acids to inhibitory exogenous saturated fatty acids also reverses inhibition. Overproduction of the ACP of fatty acid synthesis gives partial reversal of growth inhibition.

**Impact Statement**

*Enterococcus faecalis* is a major cause of hospital-acquired infections because the bacterium is hardy and resistant to many antibiotics. Knowledge of fatty acid metabolism may provide a means to inhibit *E. faecalis* infections.

## Introduction

Fatty acid biosynthesis is a highly ubiquitous cellular metabolic pathway and provides precursors for cell phospholipid synthesis, secondary metabolite production, signal molecule formation and protein post-translational modification [1]. These processes often rely on type II fatty acid synthesis (FAS II), a pathway composed of a series of discrete enzymes found in bacteria, mitochondria and plant plastids [1]. In contrast, the type I systems found in the cytosols of fungi and mammals are composed of very large multifunctional proteins (megasynthases) that contain the active sites for assembly of saturated fatty acids [2,3]. Acyl carrier protein (ACP) is essential to both pathway types because it is the carrier of the fatty acyl chains in *de novo* synthesis. ACPs also function in the incorporation of exogenous fatty acids into phospholipids [4,7].

**Fig. 1.**
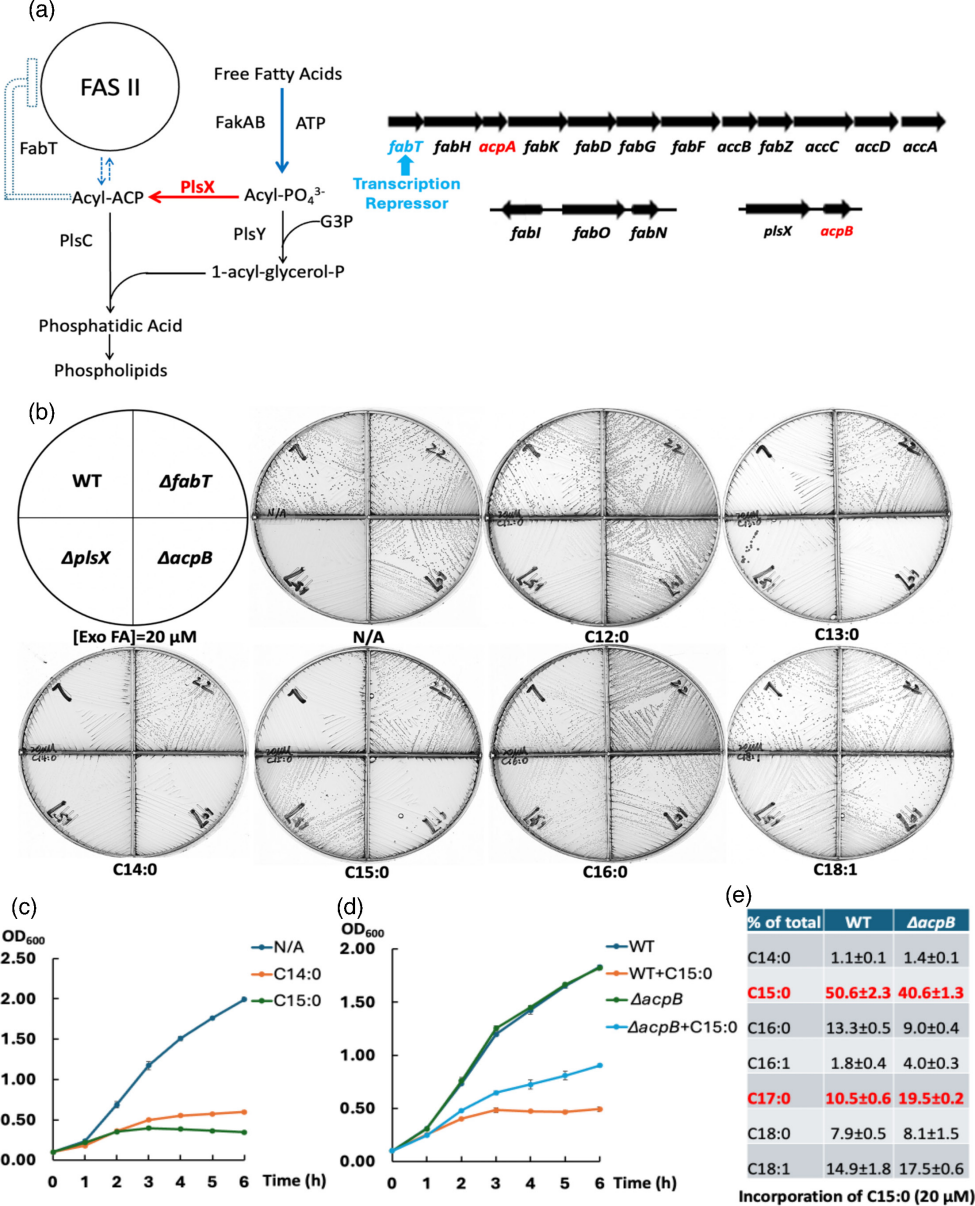
Saturated fatty acids inhibit the growth of *Enterococcus faecalis*. (a) The synthesis of phospholipids (left panel) and the genome arrangements of the fatty acid metabolism genes (right panel) of *E. faecalis*. The dashed arrows between FAS II and acyl-ACP in the left panel refer to the elongation of exogenous fatty acyl chains by *E. faecalis* FAS II. The *fabT* gene is marked with a blue arrow, and the two *acp* genes are labelled in red. (b) Growth of *E. faecalis* FA2-2 (WT), *∆fabT*, *∆acpB* and *∆plsX* strains on M17 agarose medium with various exogenous fatty acids. (c) Growth of the *E. faecalis* WT strain in the presence of myristic acid (C14 : 0) or pentadecanoic acid (C15 : 0). (d) Growth of the WT and *∆acpB* strains in the presence of pentadecanoic acid (C15 : 0). (e) GC-MS analysis of incorporation of pentadecanoic acid (C15 : 0) by *E. faecalis* WT and *∆acpB* strains. The proteins ended by the above genes are *fabD* (S-malonyltransferase), *fabG* (3-ketoacyl-ACP reductase), *acpA* (AcpA), *fabZ* (*β*-hydroxyacyl-ACP dehydratase), *fabN* (*β*-hydroxyacyl-ACP dehydratase), *fabF* (3-ketoacyl-ACP synthase II), *fabO* (3-ketoacyl-ACP synthase I), *fabI* (enoyl-ACP reductase) and *fabK* (enoyl-ACP reductase – a cryptic gene). The fatty acid synthetic pathway of *E. faecalis* is essentially that of the paradigm *Escherichia coli* pathway [48] except that *E. coli* FabA is replaced by *E. faecalis* FabN and *E. coli* FabB is replaced by *E. faecalis* FabO [24,49].

Phospholipids form the bilayer boundary between the cytoplasm and extracellular environment. Synthesis of phospholipids requires production of the central intermediate, phosphatidic acid, formed by two successive acylations of *sn*-glycerol-3-phosphate [4,5]. In addition to *de novo* synthesized fatty acyl chains, exogenous free fatty acids can be utilized as acyl chain donors [4,7]. In Gram-positive bacteria, extracellular fatty acids are first activated by a two-component fatty acid kinase (FakAB) to produce acyl phosphates [7,12], which can either acylate the *sn*-1 position of G3P via the PlsY glycerol-3-phosphate acyltransferase or be converted to acyl-ACP species by the PlsX phosphate acyltransferase. If the acyl chains are sufficiently long, acyl-ACPs acylate the *sn-2* position of lysophosphatidic acid, a reaction catalysed by the PlsC 1-acyl-*sn*-glycerol-3-phosphate acyltransferase (Fig. 1a) [5,13]. If the chains are not long enough to be phospholipid synthesis substrates, they can be elongated by the FAS II system.

However, although exogenous fatty acids are phospholipid precursors, some fatty acids are reported to have antimicrobial activity by diverse mechanisms [14]. Indeed, plants and algae produce fatty acids for protection from pathogens. Lauric acid (C12 : 0), a component of most plant seed oils (e.g. coconut), has been proven to be effective in repressing the growth of Gram-positive bacteria, whereas the monounsaturated palmitoleic acid secreted by human skin specifically disrupts the *Staphylococcus aureus* membrane [14,15]. The polyunsaturated linoleic acid is reported to alter peptidoglycan biosynthesis in Gram-positive bacteria [14]. However, the mechanisms of bacterial growth inhibition by fatty acids are unclear [14].

*Enterococcus faecalis*, a commensal Gram-positive bacterium, inhabits the gastrointestinal tract of humans. Although not considered a highly virulent micro-organism, *E. faecalis* can often lead to difficult hospital-acquired infections in surgical patients due to its resistance to multiple commonly used antibacterial agents [14,16,18].

*E. faecalis* utilizes both *de novo* synthesized and environment-derived fatty acids to form phospholipids. Detailed lipidomic analysis by Fozo and coworkers has shown that *E. faecalis* readily adapts the cell membrane lipid composition in response to environmental and genetic alterations [19,20]. The membrane lipids are phosphatidylglycerol, lysyl-phosphatidylglycerol, cardiolipin, diacylglycerol and monoglucosyldiacylglycerol, with phosphatidylglycerol being the major lipid [20,21]. The acyl chains are those common to many bacteria. The saturated acyl chains are myristic (C14 : 0), palmitic (C16 : 0) and stearic (C18 : 0) acids, whereas the unsaturated acids are palmitoleic (C16 : 1) and *cis*-vaccenic (C18 : 1) which, respectively, have their double bonds in the 9–10 and 11–12 positions. The major acyl chains are the palmitoyl and *cis*-vaccenoyl species [22,23]. However, the fatty acid composition varies with growth temperature [23]. A shortcoming of *E. faecalis* research is the lack of a chemically defined medium that supports the growth of all strains of the bacterium. Hence, the strains are grown in various commercial broth media that contain fatty acids that can be incorporated into the bacterial lipids. The plasticity of *E. faecalis* fatty acid composition is shown by mutant strains blocked in fatty acid synthesis which require an unsaturated acid for growth, whereas saturated fatty acids cannot support growth. The strains grow when provided with only oleic acid (a C18 : 1 acid with a 9,10 double bond). In the *∆acpA* strain, growth on oleic acid results in the acid comprising >99% of the membrane lipid acyl chains [24].

Most of the *de novo* fatty acid synthesis genes (the *fab* genes) are in a large operon (Fig. 1a). The first gene of the operon, *fabT*, encodes the FabT transcription repressor responsible for transcriptional regulation of the operon genes and other genes located elsewhere in the genome (Fig. 1a) [7,25,28]. *E. faecalis* encodes two ACPs, a rarity in bacteria. AcpA mediates FAS II fatty acid synthesis and is essential, whereas the nonessential AcpB functions in the incorporation of exogenous fatty acids [6,7, 24] (Fig. 1a). FabT requires an acyl-ACP ligand to activate transcriptional repression activity (Fig. 1a) [7,29]. Exogenous long-chain fatty acids (>C12) repress fatty acid synthesis through FabT and are readily incorporated into phospholipid species.

Testing the relevance of the lipid composition of *E. faecalis* in host environments is hampered by the uncertainty regarding the free fatty acid species present in the diverse environments where this bacterium is found. There certainly are free fatty acids in the gut, but the fatty acid species and levels will depend on diet and competing bacteria. In faecal-contaminated water, it is hard to say. It will depend on the level of contamination and what competitors are present. As far as infected wounds are concerned, the most prevalent *E. faecalis* infections are in root canal procedures and knee/hip replacements which may have different fatty acid contents and different competing bacteria. In this regard, a heroic and arduous undertaking by Rock and coworkers assessed the role of fatty acid uptake in wound infection by *S. aureus* using a mouse thigh wound model [30]. They found that eliminating fatty acid activation by the use of *S. aureus ΔfakA* or *ΔfakB1 ΔfakB2* strains had no effect on the growth of *S. aureus* in the wounds.

In an article by Fozo and coworkers [19] that appeared during review of this manuscript, it was noted that in *E. faecalis*, ‘the mechanism behind saturated fatty acid-induced toxicity, though, has remained unknown’. We report that exogenous saturated fatty acids at very low concentrations efficiently repress the expression of the *E. faecalis fab* genes. Inhibition of *de novo* fatty acyl chain synthesis blocks growth but is relieved by unsaturated fatty acids provided by either increased *de novo* synthesis or exogenous supplementation.

## Methods

### Materials

All fatty acids and antibiotics were purchased from Sigma-Aldrich. The media were purchased from Fisher Scientific. The DNA polymerase, restriction endonuclease, T4 ligase and Gibson Assembly Cloning Kit were purchased from New England Biolabs. Sodium [1-^14^C]acetate (specific activity, 57.0 mCi mmol^−1^) was provided by Moravek, Inc, and the [1-^14^C]lauric acid (specific activity, 55.0 mCi mmol^−1^) and [1-^14^C]palmitic acid (specific activity, 55.0 mCi mmol^−1^) were purchased from American Radiolabeled Chemicals. Silver nitrate silica gel thin-layer plates were purchased from Analtech, and Partisil KC18 plates for reverse phase (RP) chromatography were from Whatman. M17 Broth was from Becton Dickinson. All the other reagents were of the highest available quality. Oligonucleotide primers were synthesized by Integrated DNA Technologies, and DNA sequencing was performed by ACGT.

### Bacterial strains, plasmids and incubation

The bacterial strains and plasmids used in this study are listed in Table S1 (available in the online Supplementary Material), and the primers used are listed in Table S2. *Escherichia coli* cultures were grown at 37 °C in Luria–Bertani medium (tryptone, 10 g l^−1^; yeast extract, 5 g l^−1^; and NaCl, 10 g l^−1^), whereas *E. faecalis* cultures were grown at 37 °C in M17 medium (BD DIFCO). Antibiotics were added at the following concentrations (in microgram per millilitre): sodium ampicillin, 100 for *E. coli*; chloramphenicol, 30 for *E. coli* and 5 for *E. faecalis*; and erythromycin, 250 for *E. coli* and 5 for *E. faecalis*. The fatty acids were added at 20 or 100 µM unless otherwise noted.

### Construction of *E. faecalis plsY*, *plsC*, *fabD*, *fabG*, *fabF* and *fabO* expression plasmids

To construct the *E. faecalis plsY*-expression plasmid, the *plsY* gene with its promoter was amplified from *E. faecalis* FA2-2 strain genomic DNA using primer set EfPplsY *EcoRI* F and EfplsY *NcoI* R and then inserted into the *NcoI* and *EcoRI* sites of vector pQZ28 [6,7, 31, 32].

To construct the *E. faecalis plsC*-expression plasmid, the *plsC* gene was amplified from *E. faecalis* FA2-2 strain genomic DNA using primer set EfplsC *NcoI* F and EfplsC *EcoRI* R and then inserted into the *NcoI* and *EcoRI* sites of vector pQZ28 as above.

To construct the *E. faecalis fabD*-expression plasmid, the *fabD* gene was amplified from genomic DNA using primer set EffabD F and EffabD R, and the product was ligated with linearized pQZ28 vector amplified using primer set pQZ28 F1 and pQZ28-p32 R1 through Gibson assembly assay [32].

Construction of the *E. faecalis fabG*-expression plasmid was similar to the assay used for *fabD*-expression plasmid above. The *fabG* gene was amplified from *E. faecalis* FA2-2 strain genomic DNA using primer set EffabG F and EffabG R, and the product was ligated with linearized pQZ28 vector amplified using primer set pQZ28 F2 and pQZ28-p32 R2 through Gibson assembly assay.

The construction of *E. faecalis fabF* and *fabO*-expression plasmids was essentially the same as above. The encoding fragments for these two genes were amplified using primer sets EffabF *SmaI* F and EffabF *EcoRI* R, and EffabO *SmaI* F and EffabO *EcoRI* R, respectively, and then inserted into the *SmaI* and *EcoRI* sites of vector pQZ28.

### TLC analysis of radioactive labelled fatty acid methyl esters from phospholipids

To assay *de novo* synthesis of phospholipid fatty acyl chains, 5 ml *E. faecalis* cultures were inoculated at OD_600_ of 0.1 in M17 medium and incubated at 37 °C for 6 h in the presence of 1 mCi l^−1^ sodium [1-^14^C]acetate with or without a single exogenous fatty acid at the concentration given. The cells were lysed with methanol-chloroform (2 : 1) solution, and the phospholipids were extracted with chloroform and then dried under nitrogen. The fatty acyl groups were transesterified with 25% (w/v) sodium methoxide, extracted with hexanes and processed for TLC analysis on Analtech silica gel plates containing 20% silver nitrate in toluene at −20 °C. The TLC plates containing the [^14^C]-labelled fatty acid methyl esters were exposed and quantitated by phosphorimaging on a GE Typhoon FLA700 Scanner, and the data were analysed by ImageQuant TL software.

To test the incorporation of palmitic acid (C16 : 0), *E. faecalis* strains were started at OD_600_ of 0.1 in 5 ml of M17 medium, respectively, containing 0.1 mCi l^−1^ sodium [1-^14^C]palmitate plus 0.02 mM non-radioactive palmitic acid and cultured at 37 °C for 6 h. The cells were washed with PBS, and the phospholipids were extracted, methylated and analysed as described above. To test the incorporation and elongation of lauric acid (C12 : 0), *E. faecalis* strains were started at OD_600_ of 0.1 in 5 ml of M17 medium containing 0.1 µCi ml^−1^ [1-^14^C]laurate plus 0.1 mM non-radioactive lauric acid, cultured at 37 °C for 6 h and processed for extraction and esterification of fatty acyl chains on phospholipids as above. The derived fatty acyl methyl esters were analysed through reverse phase chromatography on Partisil KC18 plates of octadecyl-modified Silica Gel 60 by the solvent system of acetonitrile/methanol/water (65/35/0.5) as described previously [33]. 

Regarding the [^14^C]-labelled fatty acids, note that *E. faecalis* lacks the *β*-oxidation cycle of fatty acid degradation. This conclusion is based on *E. faecalis* genome sequences (there are no candidates for the highly conserved *β*-oxidation genes) plus the following experimental observations. Carbon isotope carboxyl-labelled fatty acids retain the label [19,20, 34]. Moreover, GC-MS of oleate-grown cells shows no evidence of the products from shortening, *cis*-7-hexadecanoate and *cis-*5-tetradecanoate. We have shown that both of these shorter acids are incorporated into lipid and readily resolved by GC-MS [32]. The same is true of the odd chain length acids. No C15 or C13 is seen in the lipids of cells fed C17 and no C13 is seen in the lipids of cells fed C15.

The lack of a *β*-oxidation pathway seems to also be the case in bacteria related to *E. faecalis*. Gullett *et al*. have fed centrally deuterated long-chain acids to *S. aureus* and *Streptococcus pneumoniae* with no evidence for chain shortening [8]. We have reported similar results in *Lactococcus lactis* [35].

### GC-MS analysis of phospholipid acyl chains

To analyse the phospholipid fatty acyl chains, *E. faecalis* strains were inoculated at an OD_600_ of 0.1 in M17 medium with or without the addition of a single fatty acid at the concentrations given and cultured at 37 °C for 6 h. The conversion of phospholipids to fatty acid methyl esters was as given for TLC analysis, and the extracted products were sent to the University of Illinois Carver Metabolomics Core for GC-MS analysis. The data were collected in triplicate.

### *β*-Galactosidase assays

The *lacZ* reporter plasmids expressing *E. coli β*-galactosidase driven by *E. faecalis fab* promoters were constructed previously [7]. *β*-Galactosidase activity was assayed as given previously [7,29, 32]. Briefly, *E. faecalis* strains transformed with *lacZ* reporter plasmids above were cultured to mid-log phase at 37 °C, and the harvested cells were washed with PBS, resuspended in Z buffer, lysed with SDS and chloroform and assayed for *β*-galactosidase activity. The data were collected in triplicate. For co-expressing *E. coli* LacZ and *E. faecalis* AcpA, the *acpA* gene was amplified from *E. faecalis* FA2-2 strain genomic DNA using primer set EfacpA *NcoI* F and EfacpA *EcoRI* R and then inserted into the NcoI and EcoRI sites of vector pZL277 [6,7, 32]. The constructed plasmids for *acpA* expression and the *lacZ* reporter plasmid were transformed into *E. faecalis* FA2-2 WT strain.

### Growth measurements of *E. faecalis* strains

To measure growth, the bacteria were started at OD_600_ of 0.1 in M17 medium at 37 °C with or without the presence of a single saturated fatty acid at 20 µM. Growth was followed for 6–8 h with readings every hour in triplicate using a Beckman DU800 spectrophotometer.

## Results

### Saturated fatty acids inhibit *E. faecalis* growth in a chain length-dependent manner

Previous work showed *E. faecalis* growth inhibition in the presence of a 100 µM concentration of a single saturated fatty acid (C12 to C15) [30]. To further examine the toxic effects of saturated fatty acids on growth, several strains were inoculated on M17 agarose plates containing various fatty acid species at 20 µM (Fig. 1b). This lower concentration was chosen because at 100 µM, lauric and pentadecanoic acids were growth inhibitory [32]. Growth of *E. faecalis* WT and *∆acpB* strains was almost totally blocked by the addition of tridecanoic acid (C13 : 0), myristic acid (C14 : 0) or pentadecanoic acid (C15 : 0), whereas growth proceeded in plates containing lauric acid (C12 : 0) or the unsaturated oleic acid (*cis*-9 C18 : 1) (Fig. 1b). Similar growth inhibition was also observed when palmitic acid (C16 : 0) was provided, although significant extents of growth on plates could be achieved upon long-term incubations (1–2 days) probably due to the depletion of the acid by its incorporation into phospholipids where it is an abundant component (Fig. 1b and data not shown).

Consistent with the solid medium results, the growth rates in liquid media of the WT strain were much slower when cultured with 20 µM myristic acid (C14 : 0), pentadecanoic acid or palmitic acid (C16 : 0), whereas the same concentration of oleic acid allowed good growth (Figs 1c, d and S1B). GC-MS analysis of the *E. faecalis* WT strain cultures incubated with myristic acid or pentadecanoic acid showed that the level of phospholipid C14 : 0 (myristoyl) chains increased by fourfold, whereas pentadecanoyl-derived chains became about 60% of the phospholipid acyl chains (Figs 1e and S2A). Note that GC-MS completely resolved the odd numbered chains from the native chains.

### Deletion of the *fabT* gene depressed growth inhibition and decreased incorporation of exogenous saturated fatty acids

**Fig. 2.**
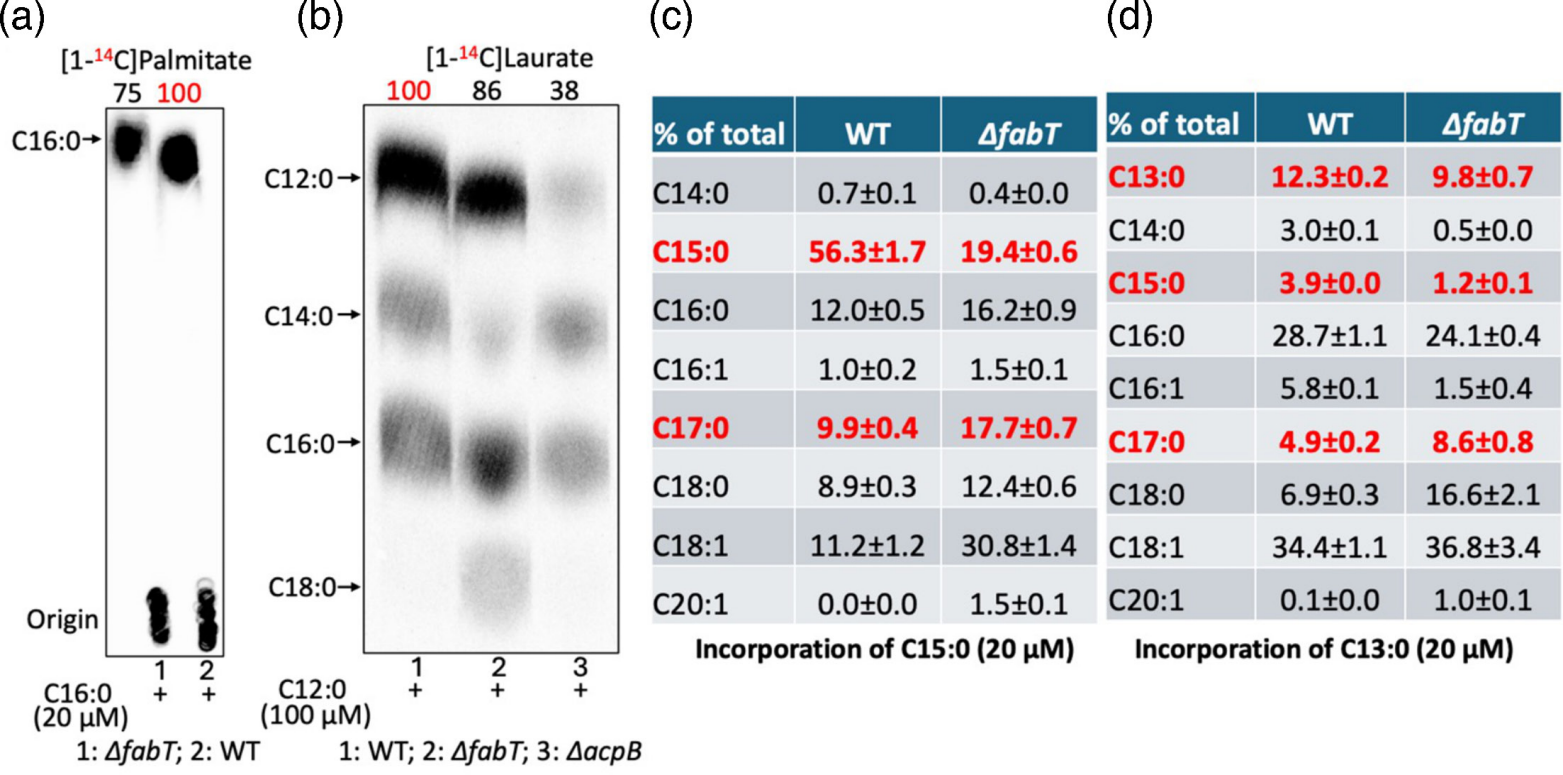
Incorporation of exogenous saturated fatty acids by the *E. faecalis ∆fabT* strain. (a) Argentation TLC analysis of [1-^14^C]palmitic acid (C16 : 0) incorporation by the *E. faecalis ∆fabT* strain. (b) RP chromatography of [1-^14^C]lauric acid incorporation by the *E. faecalis ∆fabT* strain. (c, d) GC-MS analysis of the incorporation of (**c**) pentadecanoic acid (C15 : 0) or (**d**) tridecanoic acid (C13 : 0) by the *E. faecalis ∆fabT* strain. In panels (a) and (b), the numbers above the lanes are the radioactive label incorporation values relative to the value (100) for the WT strain. In panel (a), ^14^C-labelled palmitic acid (C16 : 0) was used because GC-MS would lack sensitivity due to the high background of the acid normally present. In panel (b), ^14^C-labelled lauric acid was used to assay elongation of the acid since the elongation products are normal components (hence high backgrounds). In panels (c) and (d), GC-MS allowed the incorporation/elongation to be determined because the odd chain length acids are not normal components and are readily separated from the normal components. Note that [1-^14^C] labelled odd chain acids are not commercially available.

A striking result was that saturated fatty acids (C12-C16) failed to block the growth of an *E. faecalis ∆fabT* strain. Since this strain lacked the FabT transcriptional repressor, this indicated that growth inhibition was FabT mediated (Fig. 1b) [32]. This argued that saturated fatty acid inhibition was due to FabT repression triggered by binding acyl-ACP species derived from the exogenous acids via acyl phosphate intermediates. The role of acyl-ACP ligands was shown by the strong growth of a *∆plsX* strain in the presence of pentadecanoic acid (Fig. 1b). This strain lacked the PlsX ACP:phosphate acyltransferase required to convert acyl phosphate species to the acyl-ACP FabT ligands. The lack of repression by FabT could act to relieve saturated fatty acid toxicity by two mechanisms. The increased *de novo* synthesis could outcompete the exogenous acyl chains for incorporation into phospholipids. Another possibility is that the synthesis of unsaturated acyl chains would offset the effects of the incorporation of exogenous saturated acyl chains into phospholipids. Both mechanisms could be involved in overcoming the toxicity of a given saturated fatty acid. Indeed, increased *de novo* synthesis of the *∆fabT* resulted in decreased incorporation of [^14^C]palmitic acid (C16 : 0) and increased elongation of [^14^C]lauric acid (Fig. 2). Note that the radiolabelled fatty acids were used because their use allowed the supplemented acyl chains to be distinguished from the same acyl species synthesized *de novo*. The odd chain length acids were used because no such acyl chain species are synthesized *de novo*.

An *∆acpB* strain that lacked the fatty acid transport ACP grew more rapidly than the WT strain in the presence of either pentadecanoic acid or palmitic acid (C16 : 0) (Figs 1d and S1B). The increased growth was due to decreased repression by FabT since the incorporation of pentadecanoic acyl chains plus its elongation product was similar in WT and *∆acpB* strains. Prior work showed that acyl-AcpB species were more efficient FabT regulatory ligands than acyl-AcpA species [6,7, 29].

The relationship between toxicity and incorporation into phospholipids was measured for the native acyl chains by the use of [1-^14^C]-labelled saturated fatty acids (*E. faecalis* lacks *β*-oxidation so the acids were not shortened), whereas the incorporation of the nonnative odd chain length acids was monitored by GC-MS. This provided assays for elongation of the shorter chains to produce more effective regulatory ligands. Compared with the WT strain, the *E. faecalis ∆fabT* strain showed reduced incorporation of [1-^14^C]palmitic acid (C16 : 0) or [1-^14^C]lauric acid (C12 : 0) into phospholipids (Fig. 2a, b). Labelled lauric acid was efficiently elongated to palmitic acid (C16 : 0) and stearic acid (C18 : 0) due to the increased synthetic ability in the absence of FabT repression (Fig. 2b). Moreover, GC-MS showed more efficient elongation of the odd chain length acids, pentadecanoic acid (C15 : 0) and tridecanoic acid (C13 : 0) in the *∆fabT* strain (Fig. 2c, d). Note that acyl chains can be exchanged between AcpA and AcpB. Acyl-AcpA chains could be converted to acyl phosphates by PlsX and the acyl chains transferred to AcpB and vice versa.

### Saturated fatty acids efficiently repress *E. faecalis de novo* fatty acid synthesis

The above results demonstrated a significant role of FabT in growth inhibition by low concentrations of saturated fatty acids. This indicated that acyl-ACPs produced from exogenous saturated acids are efficient ligands for FabT repression. To assess the repression levels given by these acids following their conversion to acyl-ACP species, *β*-galactosidase (LacZ) expression from the *fabT* promoter was assayed. Note that we previously reported such data [7], but those results did not include the odd chain acids or lauric and myristic acids. Hence, some of the prior experiments were repeated to allow comparison to the results with the newly studied acids.

**Fig. 3.**
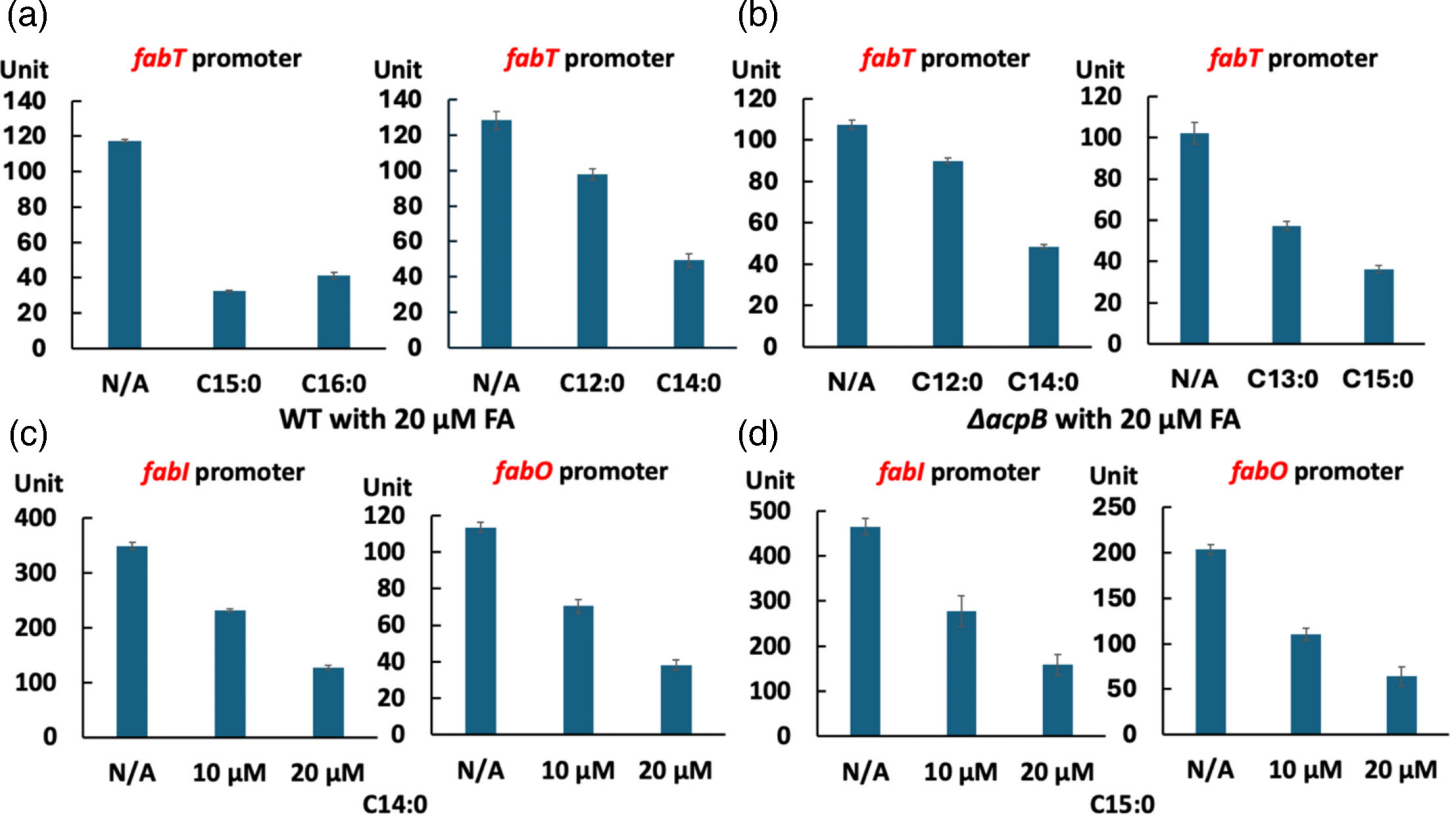
Saturated fatty acids efficiently repress the expression of the *E. faecalis fab* genes. (a, b) Expression of *β*-galactosidase from the *fabT* promoter in (**a**) *E. faecalis* WT and (b) *∆acpB* strains in the presence of various exogenous saturated fatty acid species. (c, d) Expression of *β*-galactosidase from *fabI* (at the left) or *fabO* promoters (at the right) by the *E. faecalis* WT strain in the presence of (**c**) myristic acid (C14 : 0) or (**d**) pentadecanoic acid (C15 : 0).

Supplementation with 20 µM myristic acid (C14 : 0), pentadecanoic acid (C15 : 0) or palmitic acid (C16 : 0) each resulted in a 60%–70% reduction in *β*-galactosidase activity (Fig. 3a). Weaker repression was seen with 20 µM lauric acid (C12 : 0) (Fig. 3a). Similar reductions (55%–65%) in *β*-galactosidase expression were observed for the *E. faecalis ∆acpB* strain grown with 20 µM myristic acid or pentadecanoic acid (Fig. 3b). Note that in the *∆acpB* strain, acyl-AcpA species are the default FabT repression ligand [7]. Myristic acid or pentadecanoic acid supplementation also decreased *β*-galactosidase expression from the FabT-regulated *fabI* and *fabO* promoters by 60%–70% (Fig. 3c, d).

**Fig. 4.**
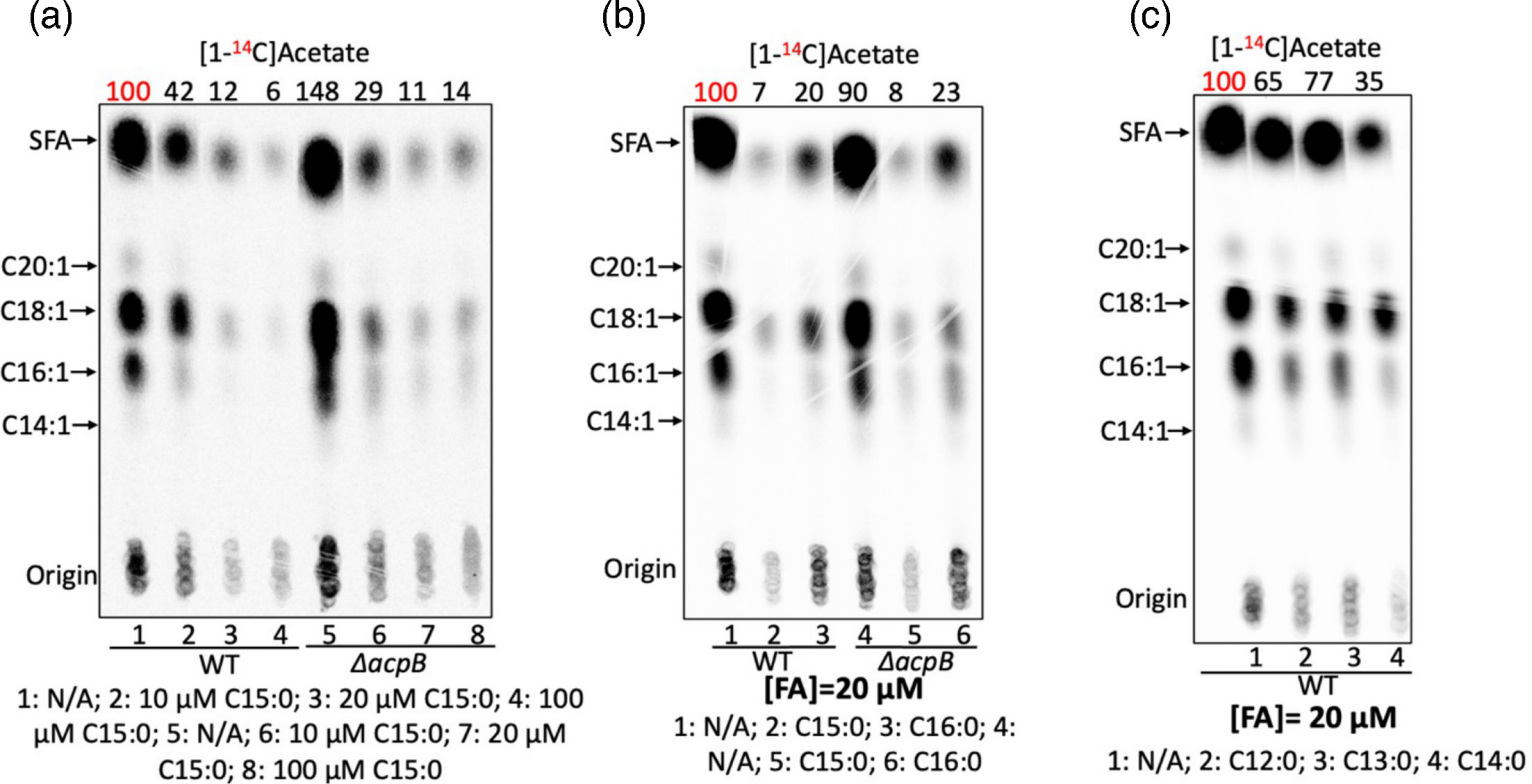
Saturated fatty acids efficiently repress *de novo* fatty acid synthesis in *E. faecalis*. (a) *De novo* fatty acid synthesis of *E. faecalis* WT and *∆acpB* strains in the presence of pentadecanoic acid (C15 : 0). (b) *De novo* fatty acid synthesis of the *E. faecalis* WT and *∆acpB* strains in the presence of palmitic acid (C16 : 0). (c) *De novo* fatty acid synthesis of *E. faecalis* WT strain in the presence of lauric acid (C12 : 0), tridecanoic acid (C13 : 0) or myristic acid (C14 : 0). In each panel, the numbers above the lanes were the radioactive label incorporation values relative to the value (100) for the WT strain cultured without exogenous fatty acids. In the case of lauric acid and tridecanoic acid (20 µM fatty acid), the cultures grew to OD_600_ values of 1.6 and 1.4, respectively, whereas without fatty acid addition, the culture grew to OD of 2.0.

The effects of saturated fatty acids on *E. faecalis de novo* fatty acid synthesis were directly assayed by [1-^14^C]acetate labelling. Surprisingly, in the *E. faecalis* WT strain, 10 or 20 µM pentadecanoic acid gave 60%–90% reductions in [1-^14^C]acetate incorporation (Fig. 4a). Consistent with the *β*-galactosidase assays above, similar efficient inhibition of acetate incorporation by pentadecanoic acid was observed in the *∆acpB* strain (Fig. 4a). In both strains, [1-^14^C]acetate incorporation was decreased by 80% when incubated with 20 µM palmitic acid (C16 : 0) (Fig. 4b), arguing that pentadecanoic acid largely mimics palmitic acid (C16 : 0). In addition, 20 µM myristic acid gave greater repression of *E. faecalis* fatty acid synthesis than lauric acid or tridecanoic acid at the same concentration (Fig. 4c), indicating that short acyl-ACPs were less effective FabT regulatory ligands. These data demonstrated that saturated fatty acids efficiently inhibit *E. faecalis* fatty acid *de novo* synthesis by FabT-dependent transcriptional repression. Note that lauric acid at 100 µM efficiently suppressed *E. faecalis fab* gene expression and inhibited *de novo* fatty acid synthesis (Fig. S5C) [32].

### Overexpression of AcpA increased *E. faecalis* resistance to saturated fatty acids

The loss of the FabT repressor increases the expression of all *fab* genes *in vivo* [6,7, 32]. To test if the expression of any single gene was dominant in overcoming saturated fatty acid toxicity, a series of plasmids expressing *fab* genes was used to overexpress each of the fatty acid synthesis proteins in the *E. faecalis* WT strain in the presence of a saturated fatty acid. The *E. faecalis* WT strain grew on plates containing 100 µM lauric acid (C12 : 0) when AcpA was overexpressed, whereas overexpression of the other fatty acid synthesis proteins had no effect (Fig. S3). The effects of AcpA overexpression were cancelled by simultaneous overproduction of the PlsX acyltransferase (Fig. S4). A possible explanation for this interaction is that AcpA overexpression would give overproduction of malonyl-AcpA, the main fatty acid synthesis substrate, whereas PlsX overexpression could result in increased levels of the acyl-ACP ligands for FabT repression and thereby render other Fab protein(s) limiting (Fig. 1a) [6,7, 32].

**Fig. 5.**
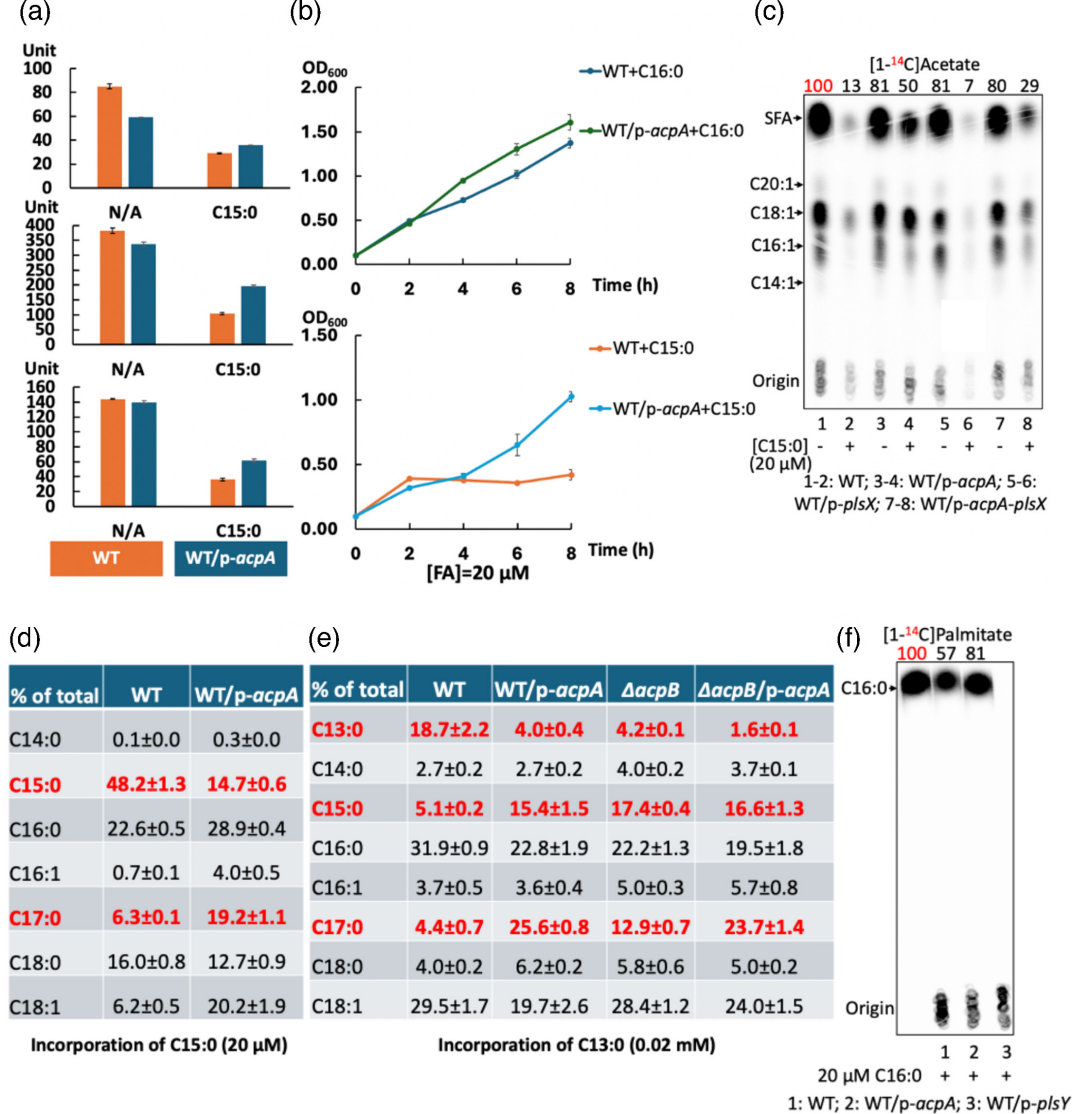
Overexpression of AcpA relieves growth inhibition by saturated fatty acids in *E. faecalis*. (a) *β*-Galactosidase expression in *E. faecalis* WT strain with *acpA*-overexpression from the *fabT* (top panel), *fabI* (middle panel) or *fabO* (bottom panel) promoters in the presence of pentadecanoic acid (C15 : 0). (b) Growth of the *E. faecalis* WT strain overexpressing AcpA in the presence of palmitic acid (C16 : 0) (top panel) or pentadecanoic acid (C15 : 0) (bottom panel). (c) *De novo* fatty acid synthesis in the *E. faecalis* WT strain overexpressing AcpA or PlsX in the presence of pentadecanoic acid (C15 : 0). (d, e) GC-MS analyses of the incorporation of (**d**) pentadecanoic acid (C15 : 0) or (**e**) tridecanoic acid (C13 : 0) by the *E. faecalis acpA*-overexpression strain. (f) Incorporation of [1-^14^C]palmitic acid (C16 : 0) by the *E. faecalis* WT strain overexpressing AcpA or PlsY. In panel (c), the numbers above the lanes are the radioactive label incorporation values relative to the value (100) for the WT strain cultured without exogenous fatty acids. In panel (f), the numbers above the lanes are the radioactive label incorporation values relative to the value (100) for the WT strain.

Interestingly, overexpression of AcpA in the presence of 20 µM pentadecanoic acid (C15 : 0) gave increased *β*-galactosidase production by the *fabT* (25%), *fabI* (90%) or *fabO* (70%) promoter fusions consistent with increased fatty acid synthesis in *E. faecalis* WT strain (Fig. 5a). Indeed, AcpA overexpression in the WT strain gave increased growth rates in the presence of either 20 µM palmitic acid or pentadecanoic acid (Fig. 5b). AcpA overexpression also gave four- to sevenfold increases in *de novo* fatty acid synthesis in both the *E. faecalis* WT and *∆acpB* strains grown with 20 µM pentadecanoic acid (Figs 5c and S5). The increased *acpA* expression stimulated the elongation of exogenous pentadecanoic acid (Fig. 5d) and tridecanoic acid (Fig. 5e), indicating that AcpA synthesis is a limiting factor in *de novo* fatty acid synthesis in these conditions.

Increased AcpA expression also resulted in a 50% enhancement in fatty acid synthesis in the WT strain cultured with 20 µM palmitic acid (C16 : 0). The increased *de novo* synthesis competed with exogenous [1-^14^C]palmitate, resulting in a >40% reduction in the incorporation of the labelled acid (Figs S5B and 5f). However, increased AcpA expression gave no significant improvement in *de novo* synthesis when myristic acid was supplied at 20 µM (Fig. S5B), probably due to the need for elongation to provide an effective regulatory ligand. The elevated level of *de novo* fatty acyl synthesis resulting from overproduction of AcpA would result in increased *de novo* synthesis of unsaturated acyl chains to form a functional phospholipid bilayer and compete with exogenous saturated acids for incorporation into phospholipid.

### Unsaturated fatty acid supplementation also overcomes growth inhibition by saturated fatty acids

**Fig. 6.**
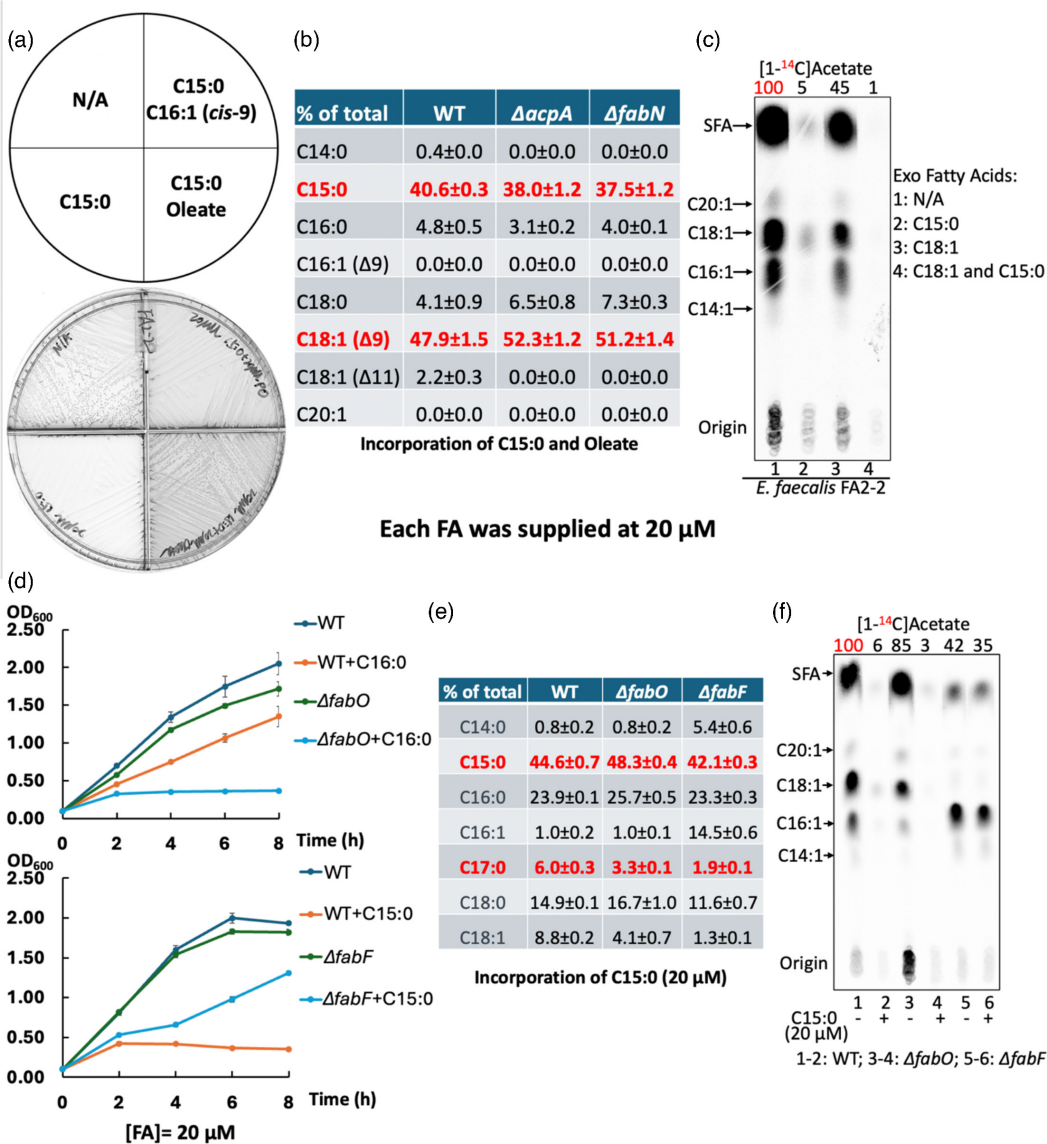
Unsaturated fatty acids protect *E. faecalis* from saturated fatty acid toxicity. (**a**) Growth of the *E. faecalis* WT strain in the presence of a mixture of equal amounts of pentadecanoic acid (C15 : 0) mixed with either oleic acid (*cis*-9 C18 : 1) or with palmitoleic acid (*cis*-9 C16 : 1) on M17 agarose medium. (**b**) GC-MS analysis of the *E. faecalis* WT and FAS II-deficient strains incorporating an equal mixture of pentadecanoic acid and oleic acid. (**c**) *De novo* fatty acid synthesis of the *E. faecalis* WT strain in the presence of oleic acid (*cis*-9 C18 : 1) or pentadecanoic acid (C15 : 0). (**d**) Growth of the *E. faecalis ∆fabO* strain in the presence of palmitic acid (C16 : 0) (top panel) and growth of the *∆fabF* strain in the presence of pentadecanoic acid (C15 : 0) (bottom panel). (e) GC-MS analysis for *E. faecalis ∆fabO* and *∆fabF* strains incorporating pentadecanoic acid (C15 : 0). (**f**) *De novo* fatty acid synthesis of *E. faecalis ∆fabO* and *∆fabF* strains in the presence of pentadecanoic acid (C15 : 0). For panels (c) and (f), the numbers above the lanes were the radioactive label incorporation values relative to the value (100) for the WT strain cultured without exogenous fatty acids.

Based on the increased synthesis of unsaturated fatty acyl chains in *∆fabT* strains, we tested the effects of oleic acid (*cis*-9 C18 : 1), palmitoleic acid (*cis*-9 C16 : 1) or tetradecenoic acid (*cis*-5 C14 : 1) on growth when the unsaturated acid was mixed with an equal amount of pentadecanoic acid (C15 : 0). When oleic acid was present, pentadecanoic acid failed to inhibit the growth of the *E. faecalis* WT strain (Fig. 6a). However, growth restoration was much weaker when palmitoleic acid was supplied (Fig. 6a) and almost disappeared in the presence of *cis*-5 tetradecenoic acid (data not shown). This may be because *cis*-5-tetradecenoic acid must be elongated for significant incorporation into phospholipid [32].

Note that oleic acid supplementation did not prevent the incorporation of pentadecanoic acid into phospholipids and resulted in only modest restoration of fatty acid synthesis (Fig. 6b, c). These data indicate that the incorporation of oleate into phospholipids per se was responsible for overcoming inhibition by pentadecanoic acid. This was consistent with prior work showing that *E. faecalis* strains that lack *de novo* fatty acid synthesis (e.g. *∆acpA*) grow well with oleic or palmitoleic acid supplements, whereas saturated fatty acids fail to support growth [24,32].

### Effects of saturated fatty acids on strains having altered de *novo* fatty acid synthesis

Mutant strains lacking either of the two long-chain 3-ketoacyl-ACP synthases, FabO and FabF, have altered fatty acid compositions [2636]. These enzymes are responsible for the elongation of acyl-ACP chains to the lengths required for phospholipid synthesis. Strains lacking FabO (*∆fabO*) have decreased contents of the two unsaturated acyl chains, C16 : 1 and C18 : 1 (palmitoleate and *cis*-vaccenate, respectively), whereas strains lacking FabF make only traces of the C18 : 1 chain and accumulate high levels of C16 : 1 phospholipid acyl chains [36]. Given the reversal of saturated fatty acid inhibition by oleate supplementation, we expected that the decreased unsaturated fatty acid synthesis ability of the *∆fabO* strain would render the strain more sensitive to inhibition by saturated fatty acids than the WT strain. This was the case. Growth of the *∆fabO* strain was almost completely blocked by 20 µM palmitic acid (C16 : 0), whereas the WT strain grew well (Fig. 6d).

In contrast, the strain lacking FabF (*∆fabF*) grew more rapidly than the WT strain when cultured with 20 µM pentadecanoic acid (Fig. 6d) and retained appreciable *de novo* fatty acid synthesis (Figs 6f and S7A, C). However, *de novo* fatty acid synthesis in the *∆fabF* strain was almost blocked when the pentadecanoic acid concentration was increased to 100 µM (Figs S2B, S7C and S8), whereas the *∆fabT* strain grew well with this concentration (Fig. S6). To our surprise, when plasmids overexpressing PlsX, PlsY or PlsC were present, growth of the *∆fabF* strain proceeded in the presence of 100 µM≥C12 acids (Figs S8 and 7). This may be due to the increased incorporation of *de novo* synthesized palmitoleic acid into phospholipids (Fig. 1a).

**Fig. 7.**
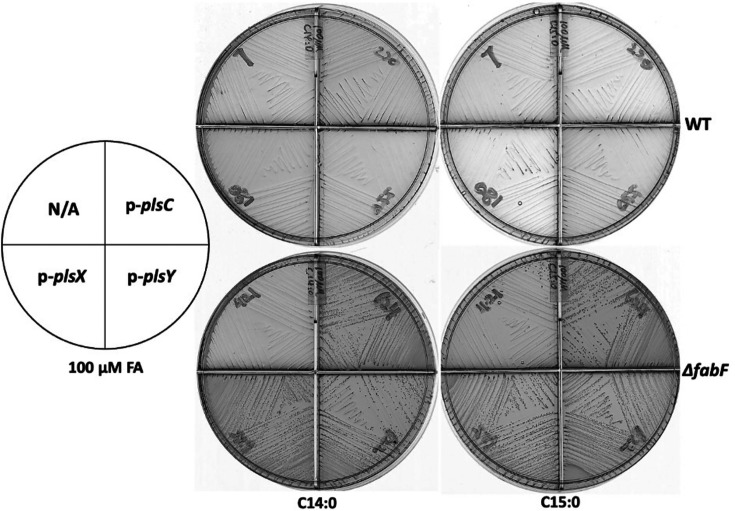
Growth of *E. faecalis ∆fabF* strain overexpressing an acyltransferase PlsX, PlsY or PlsC on M17 agarose medium in the presence of myristic acid (C14 : 0) or pentadecanoic acid (C15 : 0).

## Discussion

This report explored the physiological effects of saturated fatty acids on *E. faecalis* growth and lipid metabolism. These acids at low concentrations were found to efficiently downregulate the expression of the *fab* genes and thereby repress synthesis of unsaturated fatty acyl chains, resulting in growth inhibition (Figs1^ 3^, 4, S1 and S6). However, growth inhibition was rescued by increased *de novo* synthesis of unsaturated fatty acyl chains or by supplementation with exogenous unsaturated fatty acids (Figs1^ 6^, 7 and S8). Overexpression of AcpA partially offset growth inhibition by enhancing *de novo* unsaturated fatty acid synthesis (Figs 5, S3 and S5).

The loss of the *acpB* gene improved growth in the presence of long-chain saturated fatty acids (Figs 1 and S1) with minor effects on *fab* gene expression and fatty acid synthesis (Figs3  4). One possibility is that the deletion of *acpB* reduced the formation of acyl-ACP species and provided additional acyl phosphate for acylation of the *sn*-1 position of G3P in the initiation reaction of phospholipid synthesis (Fig. 1a). Note that AcpB rather than AcpA is the preferred acceptor of exogenous fatty acyl chains in the Fak-PlsX pathway [6,7].

Myristic acid and palmitic acid (C16 : 0) seemed less efficient than the unnatural pentadecanoic acid in repression (Figs3  4), indicating that the odd chain acid acted more like a longer acid. Palmitic acid (C16 : 0), the main saturated phospholipid acyl chain, seems to be preferred by the *E. faecalis* acyltransferases PlsY and PlsC (Fig. 1a), whereas myristic acid, a minor phospholipid component, seems less favoured (Fig. S2A). Note the possibility that in some instances, low concentrations (e.g. 20 µM) of an acid could be exhausted by incorporation into phospholipids during growth. This would terminate transcriptional repression of *fab* genes and allow *de novo* fatty acid synthesis to resume.

As we observed in the *E. faecalis ∆fabT* strain, a strain of *Streptococcus pyogenes* containing a FabT point mutation also showed resistance to growth inhibition by saturated fatty acids [37]. However, unlike the *E. faecalis ∆fabT* strain, *Streptococcus* and *Lactococcus* strains lacking FabT activity show increased synthesis of long-chain saturated fatty acids [25,26, 37]. The effects of defined fatty acid species have not been tested in *S. pyogenes.* The fatty acid sources used to study regulation and supplement mutant strains are complex mixtures: human serum or Tween 80, a nonionic detergent containing about 70% oleate with the remainder being palmitate and other chains.

The streptococci have a large 12-gene fatty acid synthesis operon with a gene arrangement that includes FabT. As seen in *E. faecalis,* FabT is a weak repressor. Upon inactivation of *S. pneumoniae*, FabT expression of the operon genes increases only three- to fivefold [26]. The *S. pneumoniae* fatty acid synthesis operon is reported to have two promoters, one upstream of FabT and a second between *acpA* and *fabK* [26]. *E. faecalis* lacks this second promoter [38,39]. Another difference between the streptococci and *E. faecalis* is in unsaturated fatty acid synthesis. *S. pneumoniae* has *fabM*, a gene located immediately upstream of *fabT* but not under FabT control, whereas *E. faecalis* has *fabN* and *fabO* located in a gene cluster distant from the large operon and expressed under FabT control [7,14]. FabM is a tetrameric enzyme that has no sequence or mechanistic similarity to FabA, a close relative of *E. faecalis* FabN [36,40,43]. The fact that these bacteria have two distinct methods to insert the double bond in unsaturated fatty acid synthesis is remarkable.

Like *E. faecalis*, the streptococci have an *acpB* gene located in a putative operon with *plsX*, and as seen in *E. faecalis*, the two genes are not regulated by FabT [44]. An *S. pyogenes ΔacpB* strain is reported to be almost completely lacking in regulation of fatty acid synthesis by exogenous fatty acids (the residual regulation is attributed to AcpA) [44]. This differs from *E. faecalis*, where the deletion of *acpB* has a less profound effect on the regulation of fatty acid synthesis by exogenous fatty acids (see above).

The *S. pneumoniae* AcpB has been studied biochemically together with the cognate FabT [45]. Electrophoretic shift experiments showed that *S. pneumoniae* FabT bound a putative operator DNA more tightly when the protein was complexed with *cis*-vaccenyl-AcpB. However, the sequence of the operator DNA used in these experiments was not given. If the sequence was the same as that used for co-crystallization with FabT, this seems concerning. This is because the FabT-operator x-ray crystal structure shows only two FabT residues that contact DNA bases, whereas the other ten contacts are with the DNA backbone phosphates [45]. Two contacts with DNA bases seem far too few to give specific binding by *S. pneumoniae* FabT, especially given the high AT content of streptococcal genomes.

Another difference between *E. faecalis* and the streptococci is that *E. faecalis* has two enoyl-ACP reductase genes *fabF* and *fabI*, whereas the streptococci have only *fabK*. Enoyl-ACP reductase catalyses the last step of the elongation cycle of FAS II pathway. The two *E. faecalis* genes encode proteins having very different structures. In *E. faecalis*, *fabK* is essentially a cryptic gene. It encodes a functional protein, but the expression fails at the level of translation [38], making FabI the functional enoyl-ACP reductase. Overproduction of *E. faecalis fabK* is lethal because it intercepts a key intermediate in unsaturated fatty acid synthesis [36]. In the streptococci, FabK and FabM compete for a common substrate [41]. Streptococci *fabM* mutants are complemented by the expression of *E. faecalis* FabN [40]. The ratio of the FabM and FabF activities must be well coordinated to allow unsaturated fatty acid synthesis [41].

*L. lactis* can be considered to be a ‘stripped down’ version of *E. faecalis* due to the genome minimization characteristic of dairy lactic acid bacteria [46]. Two *E. faecalis* genes are not present in *L. lactis*. There is no *fabK* gene in the large putative operon, and FabI (plus a putative FabN) is encoded elsewhere and seems the operative enzyme. More interesting is that there is no *acpB* gene although *L. lactis* efficiently incorporates exogenous fatty acids into its lipids [35,47]. The deletion of *L. lactis* FabT is reported to result in only a two to fourfold increase in the transcription of the operon genes [25] similar to the other FabT regulons. The *L. lactis* FabT fails to bind the *E. faecalis* FabT responsive promoters [7].

Note that the unsaturated acids, palmitoleic acid and tetradecenoic acid, may have toxic effects on *E. faecalis* that are in addition to their FabT-mediated regulatory interactions [32]. Indeed, palmitoleic acid has been reported to block specifically the growth of *S. aureus* by causing rapid membrane depolarization [15]. A caution in studying fatty acid toxicity is that at alkaline pH values, fatty acids are converted to their salts. Fatty acid salts are soaps that can have properties similar to ionic detergents.

## Supplementary material

10.1099/mic.0.001602 Uncited Fig. S1.

## Abbreviations

ACP: acyl carrier protein
FAS II: type II fatty acid synthesis
RP: reverse phase
