# Supplementary material for: Enterococcus faecalis requires unsaturated fatty acids to overcome toxicity of environmental saturated fatty acids
Source: Microbiology (Reading). 2025 Sep 3;171(9):001602. doi: 10.1099/mic.0.001602 (PMC12408189; doi:10.1099/mic.0.001602)
Supplement: Uncited Fig. S1. [file mic-171-01602-s001.pdf]

Supplement to

Enterococcus faecalis requires unsaturated fatty acids to overcome toxicity of environmental saturated fatty acids

Qi Zou, Huijuan Dong and John E. Cronan

Table S1 Strains and Plasmids

**Strains and Plasmids**	**Description**	**Source**
**Strains**		
E. faecalis FA2-2	Wild Type	Lab Strain
E. faecalis ZL116	Δ*fabT*	Zhu et al., 2019
E. faecalis QZ157	Δ*plsX*	Zou et al., 2023
E. faecalis DHJ523	Δ*acpB*	Zou et al., 2022
E. faecalis QZ243	FA2-2 with *acpA* expression plasmid (1)	Zou et al., 2024
E. faecalis QZ244	Δ*acpB* with *acpA* expression plasmid (1)	Zou et al., 2024
E. faecalis QZ180	FA2-2 with *E. faecalis plsX* expression plasmid	Zou et al., 2024
E. faecalis QZ514	FA2-2 with co-expression plasmid of *acpA* and *E. faecalis plsX*	Zou et al., 2024
E. faecalis DHJ192	Δ*fabO*	Dong & Cronan, 2022b
E. faecalis DHJ193	Δ*fabF*	Dong & Cronan, 2022b
E. faecalis QZ592	FA2-2 with *E. faecalis fabG* expression plasmid	This work
E. faecalis QZ184	Δ*acpB* with *E. faecalis plsX* expression plasmid	This work
E. faecalis QZ185	Δ*acpB* with co-expression plasmid of *acpA* and *E. faecalis plsX*	This work
E. faecalis QZ618	Δ*fabO* with *lacZ* expression plasmid from *fabT* promoter	This work
E. faecalis QZ619	Δ*fabF* with *lacZ* expression plasmid from *fabT* promoter	This work
E. faecalis QZ571	FA2-2 with *E. faecalis fabD* expression plasmid	This work

E. faecalis QZ544	FA2-2 with *E. faecalis fabF* expression plasmid	This work
E. faecalis DHJ578	FA2-2 with *E. faecalis fabO* expression plasmid	[1]
E. faecalis QZ549	FA2-2 with *E. faecalis fabN* expression plasmid	This work
E. faecalis QZ543	FA2-2 with *E. faecalis fabZ* expression plasmid	This work
E. faecalis DHJ484	Δ*acpA*	[2, 3]
E. faecalis DHJ497	Δ*fabN*	2022b
E. faecalis QZ219	FA2-2 with *lacZ* expression plasmid from *fabT* promoter	[3]
E. faecalis QZ239	FA2-2 with *lacZ* expression plasmid from *fabI* promoter	[3]
E. faecalis QZ241	FA2-2 with *lacZ* expression plasmid from *fabO* promoter	[3]
E. faecalis QZ597	FA2-2 with *lacZ* expression plasmid from *fabT* promoter and *acpA* expression plasmid (2)	This work
E. faecalis QZ604	FA2-2 with *lacZ* expression plasmid from *fabI* promoter and *acpA* expression plasmid (2)	This work
E. faecalis QZ605	FA2-2 with *lacZ* expression plasmid from *fabO* promoter and *acpA* expression plasmid (2)	This work
E. faecalis QZ190	FA2-2 with *E. faecalis fabK* expression plasmid	This work
E. faecalis QZ191	FA2-2 with *E. faecalis fabI* expression plasmid	This work
**Plasmids**		
pQZ28	Shuttled plasmid vector with a p32 promoter modified from pZL277 by replacing the chloramphenicol-resistant gene with erythromycin-resistant gene, *E. faecalis* expression	[4]
pQZ43	*E. faecalis plsX* in pQZ28	[4]
pQZ31	*E. faecalis acpA* in pQZ28 (*acpA*-expression plasmid 1)	[5]
pQZ32	*E. faecalis acpA* in pZL277 (*acpA*-expression plasmid 2)	This work
pQZ214	*E. faecalis fabT* start region (-389 to +35) at 5'-end of *lacZ* in pBHK322	[3]
pQZ235	*E. faecalis fabI* start region (-297 to +35) at 5'-end of *lacZ* in pBHK322	[3]
pQZ238	*E. faecalis fabO* start region (-297 to +35) at 5'-end of *lacZ* in pBHK322	[3]
pQZ406	*E. faecalis fabF* in pQZ28	This work
pQZ422	*E. faecalis fabZ* in pQZ28	[5]

pQZ472	pQZ28 with *acpA* and *E. faecalis plsX*	[5]
pQZ510	*E. faecalis fabD* in pQZ28	This work
pDHJ513	*E. faecalis fabG* in pQZ28	This work
pQZ379	*E. faecalis fabK* in pQZ28	[4]
pQZ387	*E. faecalis fabI* in pQZ28	[4]

Table S2 Oligonucleotides primers used in the study

**Primers***	**Sequence 5'-3'**
pQZ28 F1	CTTACTGGGAGGTAAGAATTCGTAATCATGTCATAGCT
pQZ28-p32 R1	AATCGCTGTTTTCATGGTTCACCTCCTTTTATTTTT
pQZ28 F2	GAATTCGTAATCATGTCATAGCTGTTT
pQZ28-p32 R2	GGTTCACCTCCTTTTATTTTTTTACCTAC
EfacpA NcoI F	CATGCCATGGTATTTGAAAAA
EfacpA EcoRI R	CCGGAATTCTTAGTTTGCTTGTTG
EffabG F	GTAGGTAAAAAAATAAAAGGAGGTGAACCATGGAATTAACAGGAAAAAACGT
EffabG R	AAACAGCTATGACATGATTACGAATTCTTATCCGTGCATGACTAAGC
EffabD F	AAAAGGAGGTGAACCATGAAAACAGCGATTTTATTTAGT
EffabD R	CATGATTACGAATTC TTACCTCCCAGTAAGTGC
EffabF SmaI F	TCCCCCGGGCTATGAATCGAGTAGTTATTACCG
EffabF EcoRI R	CCGGAATTCTTAATCCTCCCAGCGTTTT

* The primer sequences were based on the *E. faecalis* FA2-2 genome (NZ_CP085841.1).

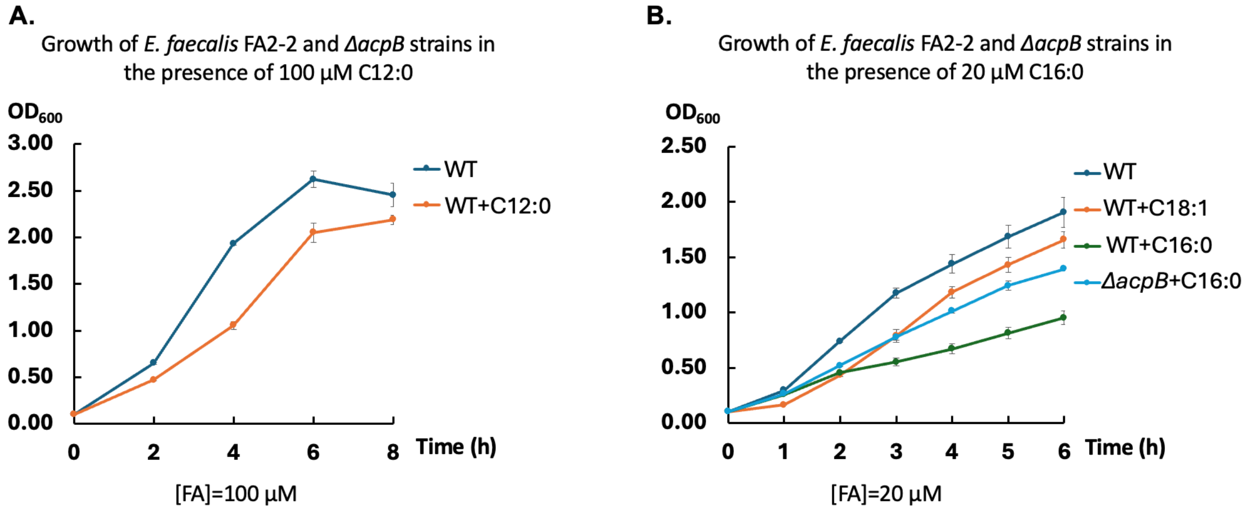

Figure S1: Growth of *E. faecalis* strains in the presence of exogenous fatty acids. **A**: Growth of *E. faecalis* wild-type strain in the presence of lauric acid (C12:0). The incubation was for 8 h and the OD_{600} values for each culture were measured every two hours in triplicate by DU800 spectrophotometer. **B**: Growth of *E. faecalis* wild-type strain in the presence of palmitic (C16:0) or oleic acid (C18:1). The incubation was for 6 hours and the OD_{600} values for each culture were measured each hour in triplicate as described above.

A.

% of total	WT	WT+C14:0
**C14:0**	**6.3±0.3**	**23.6±1.3**
C16:0	42.2±2.2	40.4±1.7
C16:1	8.9±0.5	3.7±0.3
C18:0	7.6±0.8	8.9±1.0
C18:1	34.9±2.2	23.4±1.2

Incorporation of C14:0 (20 μM)

B.

% of total	WT	Δ*fabF*
C14:0	1.0±0.6	0.8±0.1
**C15:0**	**70.0±4.8**	**82.7±1.0**
C16:0	9.3±1.9	5.1±0.3
C16:1	0.8±0.2	2.6±0.2
**C17:0**	**6.1±0.7**	**2.6±0.1**
C18:0	8.3±0.9	5.6±0.8
C18:1	4.3±2.2	0.5±0.0

Incorporation of C15:0 (100 μM)

Figure S2: Gas chromatography-mass spectrum (GC-MS) analysis of *E. faecalis* strains incorporation of various exogenous fatty acids. **A**: GC-MS analysis for *E. faecalis* wild-type strain i incorporation of myristic acid (C14:0). **B**: GC-MS for *E. faecalis* Δ*fabF* strain incorporation of pentadecanoic acid (C15:0).

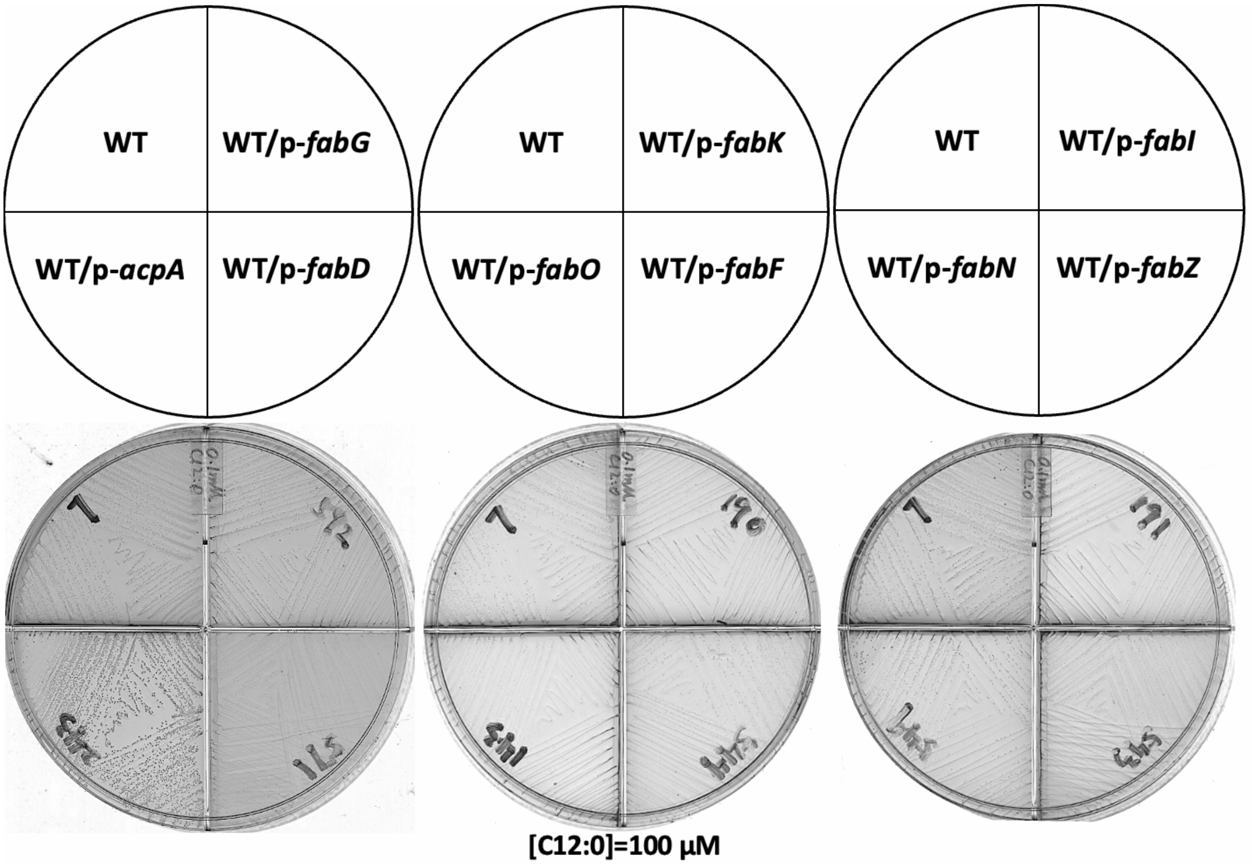

Figure S3: Growth of *E. faecalis* wild-type strain overexpressing different FAS II proteins in the presence of lauric acid (C12:0) on M17 agarose medium.

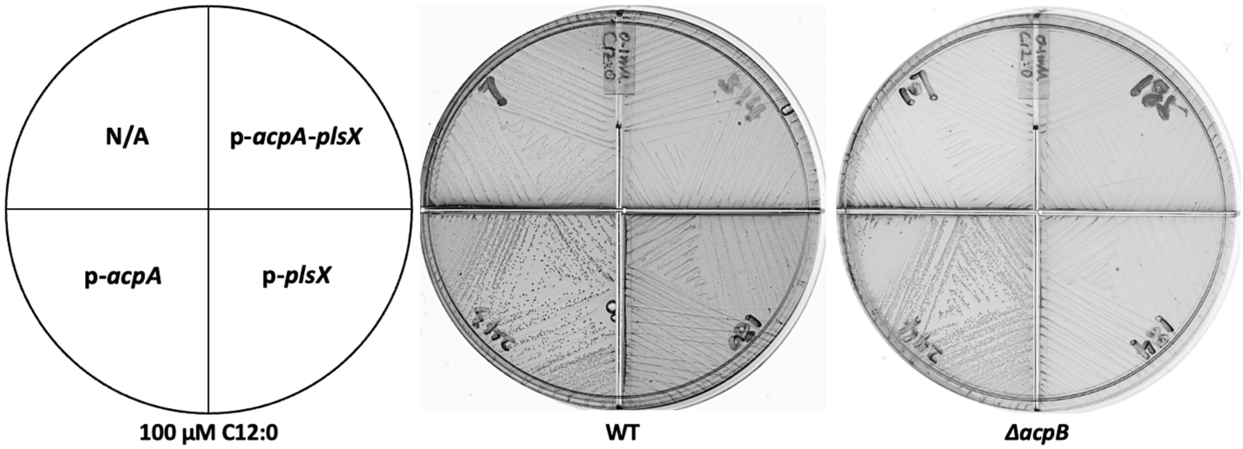

Figure S4: Growth of *E. faecalis* wild-type strain or Δ*acpB* strain overexpressing AcpA or PlsX in the presence of lauric acid (C12:0) on M17 agarose medium.

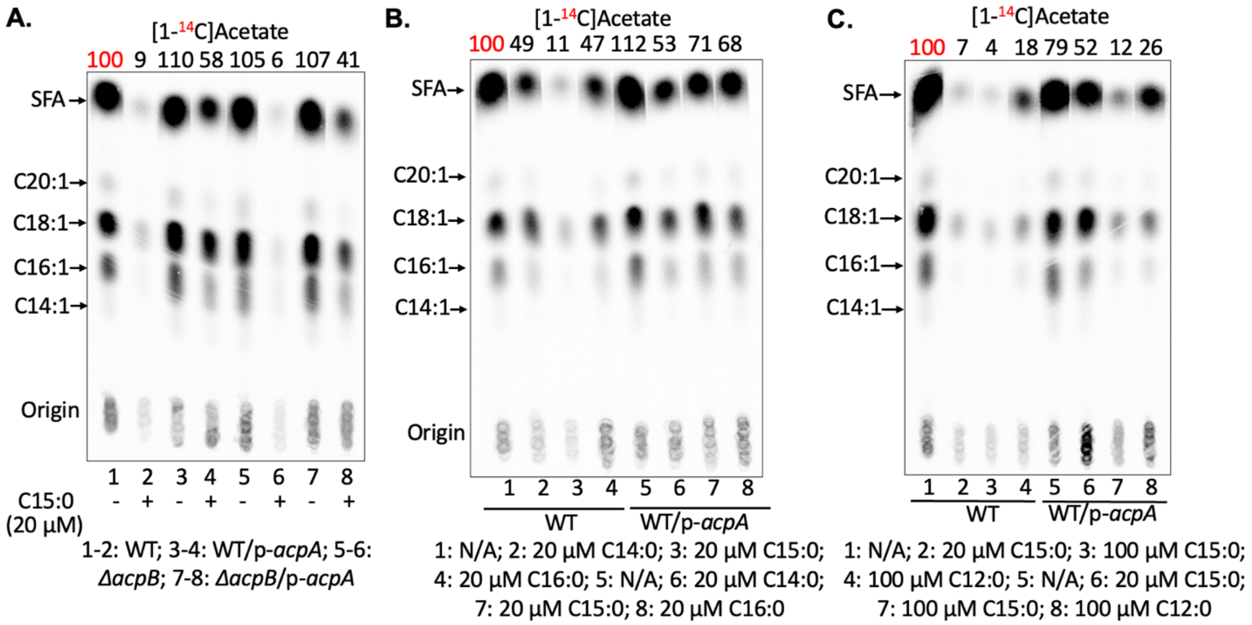

Figure S5: The effects of saturated fatty acids on *de novo* fatty acid synthesis of *E. faecalis acpA*-overexpression strains. **A**: *De novo* synthesis of fatty acids by the *E. faecalis* wild-type or Δ*acpB* strains overexpressing AcpA in the presence of pentadecanoic acid (C15:0). **B**: *De novo* synthesis of fatty acids by the *E. faecalis* wild-type strain overexpressing AcpA in the presence of various saturated fatty acid species. **C**: *De novo* synthesis of fatty acids by the *E. faecalis* wild-type strain overexpressing AcpA in the presence of pentadecanoic acid (C15:0) or lauric acid (C12:0). The numbers above the lanes were the radioactive label incorporation values relative to the value (100) for the wild-type strain cultured without exogenous fatty acids.

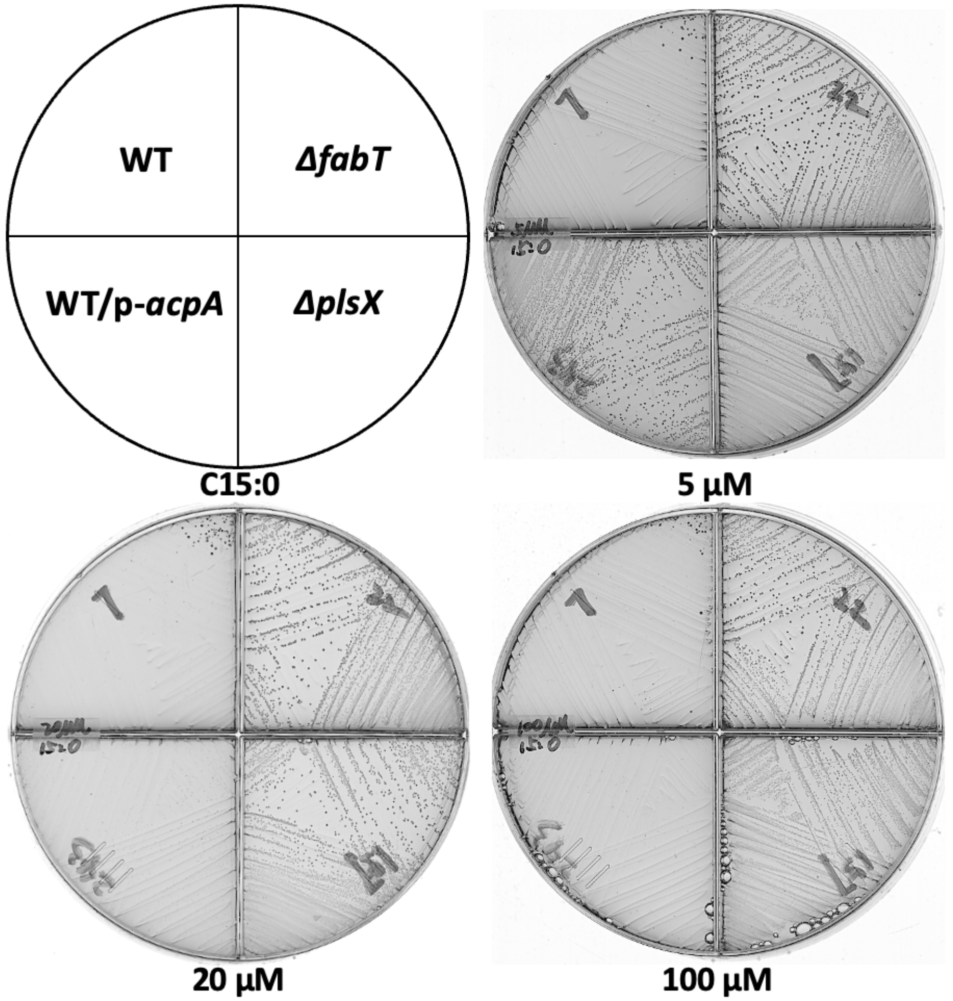

Figure S6: Growth of the *E. faecalis* Δ*fabT* and wild-type strains overexpressing AcpA in the presence of various concentration of pentadecanoic acid (C15:0) on M17 agarose medium.

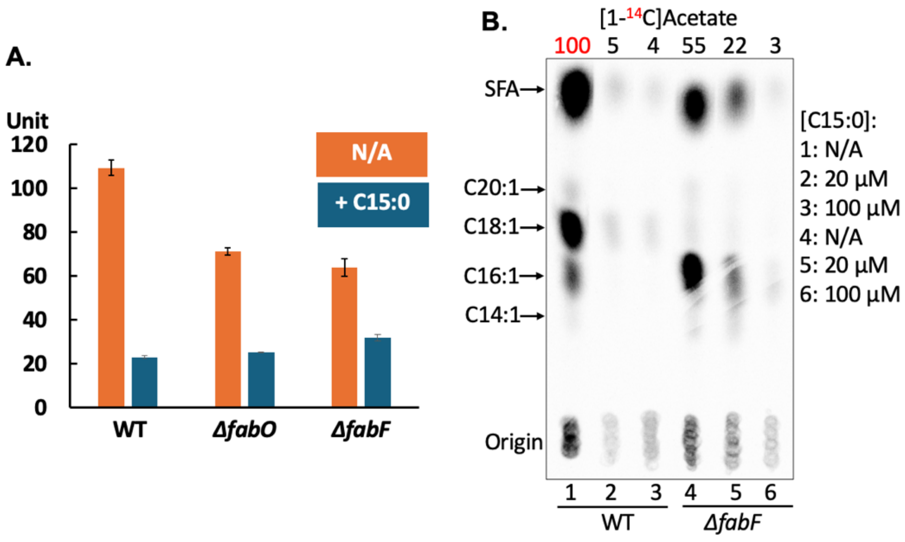

Figure S7: *E. faecalis* Δ*fabF* strain contained resistance to saturated fatty acids. **A**: Expression of β-galactosidase from the *fabT* promoter by *E. faecalis* Δ*fabO* or Δ*fabF* strain in the presence of pentadecanoic acid (C15:0). **B**: *De novo* synthesis of fatty acids by *E. faecalis* Δ*fabF* strain in the presence of various concentration of pentadecanoic acid. In panel **B**, the numbers above the lanes were the radioactive label incorporation values relative to the value (100) for the wild-type strain cultured without exogenous fatty acids.

Supplementary references

1. **Dong H, Cronan JE**. Unsaturated fatty acid synthesis in *Enterococcus faecalis* requires a specific enoyl-ACP reductase. *Mol Microbiol* 2022;118:541-551.
2. **Dong H, Cronan JE**. The two acyl carrier proteins of *Enterococcus faecalis* have nonredundant functions. *J Bacteriol* 2022;204:e0020222.
3. **Zou Q, Dong H, Zhu L, Cronan JE**. The *Enterococcus faecalis* FabT transcription factor regulates fatty acid biosynthesis in response to exogeneous fatty acids. *Front Microbiol* 2022;13:877582.
4. **Zou Q, Dong H, Cronan JE**. Growth of *Enterococcus faecalis* Δ*plsX* strains is restored by increased saturated fatty acid synthesis. *mSphere* 2023;8:e0012023.
5. **Zou Q, Dong H, Cronan JE**. The enteric bacterium *Enterococcus faecalis* elongates and incorporates exogenous short and medium chain fatty acids into membrane lipids. *Mol Microbiol* 2024;122:757-771.
